# Lipid nanoparticles for mRNA therapy: recent advances in targeted delivery

**DOI:** 10.1093/lifemedi/lnac004

**Published:** 2022-06-24

**Authors:** Tuo Wei, Wei Tao, Qiang Cheng

**Affiliations:** State Key Laboratory of Stem Cell and Reproductive Biology, Institute of Zoology, Chinese Academy of Sciences, Beijing 100101, China; Beijing Institute for Stem Cell and Regenerative Medicine, Beijing 100101, China; Institute for Stem Cell and Regeneration, Chinese Academy of Sciences, Beijing 100101, China; University of Chinese Academy of Sciences, Beijing 100049, China; Center for Nanomedicine and Department of Anesthesiology, Brigham and Women’s Hospital, Harvard Medical School, Boston, Massachusetts 02115, United States; Department of Biomedical Engineering, College of Future Technology, Peking University, Beijing 100871, China

The approval of two mRNA vaccines against coronavirus disease 2019, BNT162b and mRNA-1273, has accelerated progress and development of mRNA medicines that have now reached the general public worldwide. Since the first discovery of mRNA in 1960s, researchers have been working consistently to explore the possibility of mRNA drugs for diseases therapy, but success was largely limited by their instability and immunogenicity. Rapid development of mRNA engineering strategies made it possible to overcome these drawbacks [1]. Through optimization of the untranslated regions (3ʹUTR and 5ʹUTR), caps, poly(A) tails, and codons of the open reading frame, the stability and translational efficiency of *in vitro* transcribed mRNA was significantly improved. Incorporation of chemically modified nucleosides (such as 5-methyluridine and N^1^-methylpseudouridine) further reduced immunogenicity and increased stability. All of such engineering strategies accelerated the clinical translation of mRNA drugs.

As naked mRNA could hardly cross the cell membrane, many types of delivery carriers have been developed, including lipid-based nanoparticles, polymer-based nanoparticles, peptide-based vehicles, and protein-mRNA complexes. Among them, lipid nanoparticles (LNPs) are currently the most advanced vectors. Typically, LNPs contain four lipid components. Ionizable cationic lipid is the most important component and essential for delivery potency; amphipathic phospholipid helps to encapsulate and release cargos; cholesterol assists for stability of formulations; and PEGylated lipid regulates stability and circulation time of LNPs in the blood. The structure and internal ratios of these components play important roles on mRNA delivery. By library screening of lipid structures and optimization of internal ratios, a variety of LNPs have been developed for mRNA applications in multiple areas, including vaccines, protein replacement, gene editing, and cell therapy.

mRNA is required to be delivered into specific organs or cells to satisfy therapy. Surface tissues (e.g. muscle and eyes) are readily reached by local administration. But local delivery of mRNA LNPs to some organs that require effective penetration and long-term retention remains difficult. We recently developed mucoadhesive nanoparticles for intravesical delivery of lysine-specific demethylase 6A (KDM6A) mRNA to overcome physiological bladder barriers for treatment of bladder cancer metastasis [2]. For organs deep in the body that are hardly achieved by local delivery, systemic administration is more favorable. However, LNPs tend to accumulate in liver after intravenous injection, making extrahepatic mRNA delivery very challenging. Recently, several advanced delivery strategies have been reported for targeted mRNA delivery beyond the liver.

**Figure 1.**
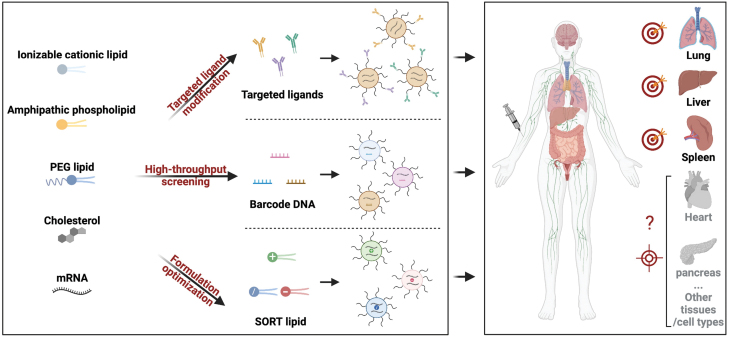
Illustration showing advanced strategies for *in vivo* mRNA delivery beyond the liver. Targeted ligand modification, high-throughput screening, and formulation optimization accelerated the development of LNPs for tissue targeted delivery of mRNA *in vivo*.

Functionalization of targeting moieties on LNPs is a straightforward way to enhance cell tropism (Fig. 1). A recent study using *in vivo* chimeric antigen receptor (CAR) T cells to treat cardiac injury chose this strategy [3]. They conjugated anti-CD5 antibody onto LNPs (CD5/LNPs) to deliver mRNA encoding a CAR against fibroblast activation protein (FAP). This system could target splenic T cells that overexpress CD5 and produce FAPCAR^+^ T cells in mice. These *in vivo* generated FAPCAR^+^ T cells reduced fibrosis and improved cardiac function in a mouse model of cardiac injury. Similarly, Dan Peer and coworkers conjugated a recombinant fusion protein (MadCAM-1-D1D2-Fc) onto LNPs through a secondary antibody against rat IgG_2a_ (termed RG7), which could recognize a specific protein conformation of integrin α4β7 expressed on gut-homing leukocytes and enabled targeted delivery to a selective subset of leukocytes, showing therapeutic potential in a mouse model of colitis [4]. Different from antibody targeting, Gabriel A. Kwong and coworkers recently developed a UV light induced ligand exchange platform of MHC class I antigen peptides that can be post-inserted into LNPs. This system allows rapid switch of MHC class I antigen peptides for targeted mRNA delivery into multiple populations of antigen specific T cells *in vivo* [5]. Such modifications of LNPs provide a fast and rational strategy for enhanced cell targeting, especially for immune cells in the blood.

Library screening of lipid structures is already proven an effective way to identify LNPs targeting non-liver tissues [6]. To improve the screening efficacy, James Dahlman and coworkers developed a high-throughput DNA barcoding system, named FIND [7]. They formulated a variety of LNPs encapsulating Cre mRNA and a unique DNA barcode in each LNP. These LNPs were intravenously injected into Cre reporter mice and then tdTomato-positive cells were isolated using FACS followed by DNA sequencing to identify LNPs (Fig. 1). Using this system, they quantified over 250 LNPs simultaneously and found two lead LNPs targeting splenic endothelial cells.

To seek a more predictable way for extrahepatic delivery, we developed a methodology (SORT), which enabled mRNA translation specifically in mouse liver, spleen, or lungs by simply adding a supplemental SORT molecule into traditional LNP components [8] (Fig. 1). By increasing the percentage of a cationic lipid incorporated, the protein expression moved gradually from liver to spleen, and then to lungs, showing a clear and precise tissue tropism *in vivo*. Inclusion of an anionic lipid enabled specific delivery of mRNA to the spleen, while inclusion of an ionizable cationic lipid aided in improved liver targeting. Importantly, the SORT technology could help reengineering other types of LNPs to realize organ specific delivery of mRNA [9], providing a guideline of LNP designing for tissue-specific mRNA delivery. The mechanism study revealed that SORT molecules controlled the biodistribution, global p*K*_a_, and plasma protein absorption of SORT LNPs that eventually decided the LNPs fate *in vivo* [10].

It is noted that the absorption of plasma proteins onto LNPs may play a key role on tissue targeting. Identifying these unique proteins is very important and may give us more inspiration on developing new LNPs to target other organs, such as heart, brain, and pancreas. Moving forward, achieving cell-specific mRNA delivery in targeted tissues is more challenging but critical, which may accelerate the broad applications of RNA therapeutics in precision medicine. Combing tissue and cell targeting strategies may be a good way, but there are still concerns needed to figure out. For example, ligand/antibody modified LNPs may disrupt the balance of internal components and recruitment of protein corona in blood, which may affect the established tissue specificity of LNPs. With more depth studies focused on this field, we believe more precise targeting strategies will be emerged and applied for precise medicine in the future.
